# Phenotypic and metabolic adaptations of *Rhodococcus cerastii* strain IEGM 1243 to separate and combined effects of diclofenac and ibuprofen

**DOI:** 10.3389/fmicb.2023.1275553

**Published:** 2023-12-06

**Authors:** Elena Tyumina, Grigory Bazhutin, Nadezhda Kostrikina, Vladimir Sorokin, Andrey Mulyukin, Irina Ivshina

**Affiliations:** ^1^Perm Federal Research Center, Ural Branch of the Russian Academy of Sciences, Institute of Ecology and Genetics of Microorganisms, Perm, Russia; ^2^Department of Microbiology and Immunology, Perm State University, Perm, Russia; ^3^Winogradsky Institute of Microbiology, Research Center of Biotechnology, Russian Academy of Sciences, Moscow, Russia

**Keywords:** rhodococci, NSAIDs, ibuprofen, diclofenac, stress responses, AFM/CLSM, TEM, TEM-EDX

## Abstract

**Introduction:**

The increasing use of non-steroidal anti-inflammatory drugs (NSAIDs) has raised concerns regarding their environmental impact. To address this, understanding the effects of NSAIDs on bacteria is crucial for bioremediation efforts in pharmaceutical-contaminated environments. The primary challenge in breaking down persistent compounds lies not in the biochemical pathways but in capacity of bacteria to surmount stressors.

**Methods:**

In this study, we examined the biodegradative activity, morphological and physiological changes, and ultrastructural adaptations of *Rhodococcus cerastii* strain IEGM 1243 when exposed to ibuprofen, diclofenac, and their mixture.

**Results and Discussion:**

Our findings revealed that *R. cerastii* IEGM 1243 exhibited moderate biodegradative activity towards the tested NSAIDs. Cellular respiration assay showed higher metabolic activity in the presence of NSAIDs, indicating their influence on bacterial metabolism. Furthermore, catalase activity in *R. cerastii* IEGM 1243 exposed to NSAIDs showed an initial decrease followed by fluctuations, with the most significant changes observed in the presence of DCF and the NSAID mixture, likely influenced by bacterial growth phases, active NSAID degradation, and the formation of multicellular aggregates, suggesting potential intercellular synergy and task distribution within the bacterial community. Morphometric analysis demonstrated alterations in size, shape, and surface roughness of cells exposed to NSAIDs, with a decrease in surface area and volume, and an increase in surface area-to-volume ratio (SA/V). Moreover, for the first time, transmission electron microscopy confirmed the presence of lipid inclusions, polyphosphates, and intracellular membrane-like structures in the ibuprofen-treated cells.

**Conclusion:**

These results provide valuable insights into the adaptive responses of *R. cerastii* IEGM 1243 to NSAIDs, shedding light on the possible interaction between bacteria and pharmaceutical compounds in the environment.

## 1 Introduction

Chemical pollution of the environment, alongside climate change and catastrophic biodiversity loss, is recognized as a major global environmental problem demanding urgent attention and resolution (Wang et al., 2021; Brack et al., 2022). Of particular concern is the continuous introduction of active pharmaceutical ingredients (APIs) into ecosystems. Due to their biological activity and stable chemical structure, APIs have adverse effects on non-target organisms and pose a significant threat to the delicate balance of aquatic and terrestrial environments (Mulkiewicz et al., 2021; Wilkinson et al., 2022).

Among the various APIs detected in environmental samples, nonsteroidal anti-inflammatory drugs (NSAIDs) have garnered considerable attention owing to their widespread use and persistence (Richards et al., 2011; Domaradzka et al., 2015; Izadi et al., 2020; Mejía-García et al., 2020; Parolini, 2020; Herzig et al., 2021; Mulkiewicz et al., 2021; Petrie and Camacho-Muñoz, 2021; Rastogi et al., 2021; Wojcieszyńska et al., 2022). Specifically, diclofenac (DCF) and ibuprofen (IBP), which consistently rank among the most frequently detected pharmaceuticals in environmental matrices, have drawn considerable interest. The incomplete decomposition of DCF and IBP in the human body leads to their migration through sewage and wastewater treatment plants to surface water (Lonappan et al., 2016; Chopra and Kumar, 2020). The improper disposal of unused or expired DCF and IBP significantly contributes to their environmental contamination, as these medications are often flushed down toilets or disposed of in landfills, ultimately entering wastewater treatment plants or leaching into groundwater (Sadutto et al., 2021). Additionally, the manufacturing process of DCF and IBP can release these compounds into the environment, further contributing to their presence in ecosystems (Bibi et al., 2023).

The main environmental hazard of these NSAIDs lies in their biological activity, even at low (μg/L) concentrations. The toxic effects of DCF and IBP on birds, aquatic vertebrates and invertebrates, microalgae, and bacterial consortia have been well-documented (Cleuvers, 2004; Swan et al., 2006; Ericson et al., 2010; Parolini et al., 2011; De Felice et al., 2012; Memmert et al., 2013; González-González et al., 2014; Gamarra et al., 2015; Davids et al., 2017; Mezzelani et al., 2018; Aguilar-Romero et al., 2020; Chopra and Kumar, 2020; Parolini, 2020; Jan-Roblero and Cruz-Maya, 2023). Exposure to these NSAIDs can disrupt reproductive processes in living organisms, impair growth, and even cause mortality (Ortiz de García et al., 2014; Lonappan et al., 2016). DCF and IBP have the potential to bioaccumulate in the food chain, meaning they can concentrate in the tissues of organisms at higher levels than in the surrounding environment (Xie et al., 2015; Mezzelani et al., 2018). This accumulation can lead to adverse effects in higher trophic levels, including fish-eating birds and mammals. Moreover, the toxicity of DCF and IBP can have cascading effects on aquatic ecosystems, altering the balance of species and disrupting crucial ecosystem functions (Świacka et al., 2021).

In addition to addressing the environmental impact of these NSAIDs, extensive research has elucidated the microbial biodegradation pathways of NSAIDs, as comprehensively reviewed by Guzik and Wojcieszyńska (2019). However, despite significant efforts to examine the effects of NSAIDs on various organisms, our understanding of their specific impacts on microorganisms, particularly with respect to response mechanisms and adaptation patterns, remains limited (Jiang et al., 2017; Ivshina et al., 2019; Tyumina et al., 2019, 2020; Żur et al., 2020; Ivshina et al., 2021b; Żur et al., 2021).

Rhodococci are intriguing members of microbial communities, renowned for their remarkable survival strategies and exceptional ability to metabolize xenobiotic compounds, including pharmaceuticals (Yam et al., 2010; de Carvalho, 2012; Cheremnykh et al., 2018; Kuyukina and Ivshina, 2019; Rodrigues and de Carvalho, 2019; Pátek et al., 2021; Ivshina et al., 2021a, 2022c). These survival mechanisms encompass cell aggregation, production of extracellular polymeric substances, changes in lipid content, reduction of reactive oxygen species, and accumulation of intracellular storage compounds (Pátek et al., 2021). Despite their potential as valuable bioremediation agents, our understanding of how rhodococci respond to pharmaceuticals, particularly NSAIDs, remains scarce.

In our previous studies, we provided evidence of the biodegradation potential of rhodococci, demonstrating their ability to degrade NSAIDs such as DCF, paracetamol, IBP, and ketoprofen (Ivshina et al., 2006, 2019, 2021b; Bazhutin et al., 2022). Building on these foundational investigations, our current research aims to explore the individual and combined effects of IBP and DCF on *Rhodococcus cerastii* strain IEGM 1243 (GenBank # JAJNDD010000001-JAJNDD010000295; http://www.iegmcol.ru/strains/rhodoc/cerastii/r_cerastii1243.html accessed on 31 May 2023). Understanding the response mechanisms and adaptive behavior of rhodococci to pharmaceutical exposure is vital for assessing the potential risks posed by the widespread occurrence of NSAIDs and for developing effective strategies to mitigate their environmental impact.

In light of the aforementioned gaps in knowledge, this investigation aims to address the following research questions: (1) How do NSAIDs, specifically IBP and DCF, affect the biodegradative activity of *R. cerastii* strain IEGM 1243? (2) What are the morphological and physiological changes in *R. cerastii* IEGM 1243 cells when exposed to IBP, DCF, and their mixture? (3) Are there ultrastructural adaptations in *R. cerastii* IEGM 1243 cells in response to NSAID exposure?

This investigation contributes to the expanding body of knowledge concerning the ecotoxicity of pharmaceuticals and the intricate interactions between microorganisms and environmental contaminants. The findings of this study will provide important information on the effect of NSAIDs on rhodococci. Understanding how biodegraders adapt can help to build more efficient biocatalysts, allowing for more informed decision-making and encouraging sustainable management of pharmaceutical pollution in ecosystems.

## 2 Materials and methods

### 2.1 Strain cultivation

*R. cerastii* strain IEGM 1243 from the Regional Specialized Collection of Alkanotrophic Microorganisms (IEGM; WFCC-WDCM 768; UNU/CKP 73559/480868; www.iegmcol.ru accessed on 31 May 2023) was used in this study. The strain IEGM 1243 was isolated from soil, lake shore Kumnylor, Tyumen region, Russia. In preliminary studies it showed IBP-degrading (100 mg/L) activity in the presence of 0.1% *n*-hexadecane (data not shown). Moreover, IEGM 1243 uses *n*-hexadecane as a sole carbon source and is resistant to Mo^6+^ (5.0 mM). The experiments were conducted in 250 mL Erlenmeyer flasks containing 100 mL of “RS” (*Rhodococcus* Surfactant) mineral salt medium. The composition of the medium per liter included: KNO_3_ 1.0 g, K_2_HPO_4_ 1.0 g, KH_2_PO_4_ 1.0 g, NaCl 1.0 g, MgSO_4_ × 7H_2_O 0.2 g, CaCl_2_ × 2H_2_O 0.02 g, FeCl_3_ 0.001 g, and 0.1% (v/v) trace element solution (1.5 g/L FeCl_3_ × 7H_2_O, 0.1 g/L H_3_BO_3_, 0.01 g/L ZnSO_4_ × 7H_2_O, 0.05 g/L Co (NO_3_)_2_ × 6H_2_O, 0.005 g/L CuSO_4_ × 5H_2_O, and 0.005 g/L MnCl_2_ × 4H_2_O). IBP (C_13_H_17_O_2_Na; CAS: 31121-93-4; (RS)-2-(4-(2-methylpropyl)phenyl)propanoic acid) and DCF (C_14_H_10_Cl_2_NNaO_2_, CAS: 15307–79-6, 2-(2-(2,6-dichlorophenylamino)phenyl)acetic acid) were used as sodium salts (Sigma-Aldrich, St. Louis, MO, United States; Glentham Life Sciences, Corsham, United Kingdom) and added to the mineral salt medium, each at a final concentration of 50 mg/L. Stock solutions of IBP and DCF (50 mg/mL) were prepared individually using water and stored at 4°C before use. We opted for this high concentration of NSAIDs due to the potential application of *Rhodococcus*-based biocatalysts in local post-treatment technologies during drug manufacturing, where influents contain elevated pharmaceutical levels. Moreover, previous research on biodegradation and the diverse effects of pharmaceuticals on bacteria supports the use of higher dosages (Alobaidi et al., 2021; Żur et al., 2021; Mohamed et al., 2023; Suleiman et al., 2023). D-glucose was employed as an additional carbon and energy source at a concentration of 0.5 g/L. Cells of *R. cerastii* IEGM 1243 grown for 3 days in LB broth (Himedia Laboratories Pvt. Limited, Maharashtra, India) and washed twice with phosphate buffer (pH 7.0) were added to the medium to a concentration of 0.96 × 10^7^ cells/mL. The experiments were conducted at 160 rpm and 28°C.

The following treatments were used in the experiments: (1) mineral salt medium + *R. cerastii* IEGM 1243 + 0.5 g/L glucose (biotic control), (2) mineral salt medium + *R. cerastii* IEGM 1243 + 0.5 g/L glucose + 50 mg/L IBP (positive control 1), (3) mineral salt medium + *R. cerastii* IEGM 1243 + 0.5 g/L glucose +50 mg/L DCF (positive control 2), (4) mineral salt medium + *R. cerastii* IEGM 1243 + 0.5 g/L glucose + NSAID mixture (50 mg/L IBP + 50 mg/L DCF) (positive control 3), (5) mineral salt medium +0.5 g/L glucose + 50 mg/L NSAID (abiotic controls), (6) mineral salt medium + autoclaved *R. cerastii* IEGM 1243 + 0.5 g/L glucose + 50 mg/L NSAID (sorption controls). The abiotic controls were necessary to elucidate the abiotic degradation of the pharmaceuticals, while the sorption controls were essential for assessing the removal of the pharmaceuticals by sorbing them onto bacterial cells. The biotic (negative) control was essential to differentiate the effects of NSAIDs on bacterial cells from the natural variations in their life cycle.

### 2.2 Microscopic investigations

#### 2.2.1 Cell viability

To determine the viability of cells, the bacterial suspension was stained using a LIVE/DEAD^®^ *Bac*Light^™^ Bacterial Viability Kit following the manufacturer’s protocol (Invitrogen, Waltham, MA, United States). The staining process allowed for differentiation between live and dead cells. Visualization of the stained cells was carried out using an Axio Imager M2 microscope (Carl Zeiss Microscopy GmbH, Jena, Germany) in fluorescence mode (Luchnikova et al., 2022). Images were captured by an Axoicam 506 Color camera (Carl Zeiss Microscopy GmbH, Jena, Germany) and Zen Blue 3.1 software (Carl Zeiss Microscopy GmbH, Jena, Germany).

#### 2.2.2 Lipid inclusions staining

To identify intracellular lipid inclusions, the rhodococci were stained with Nile Red (Nanjing Dulai Biotechnology Co., Nanjing, China) (Spiekermann et al., 1999) with slight modifications (Luchnikova et al., 2022). Briefly, the cell suspension was centrifuged at 12,000 rpm for 5 min to obtain a cell pellet. The cell pellet was then resuspended in 1 mL of distilled water and mixed with 40 μL of a 0.08% solution of Nile Red in dimethyl sulfoxide. This suspension was incubated at 28°C with shaking at 160 rpm for 40 min. After incubation, cells were harvested by centrifugation (12,000 rpm for 5 min), resuspended in 1 mL of distilled water and visualized using the Axio Imager M2 microscope (Carl Zeiss Microscopy GmbH, Jena, Germany). Two different spectral settings were utilized: yellow-gold fluorescence with a 450–500 nm band pass exciter filter and red fluorescence with a 515–560 nm band pass exciter filter.

#### 2.2.3 Surface topography and nanostructure of bacterial cells

The influence of NSAIDs on the morphology and surface topography of cells was investigated using a combined scanning system, comprising an atomic force microscope (AFM) MFP-3D-BIO^™^ (Asylum Research Inc., Santa Barbara, CA, United States) and a confocal laser scanning microscope (CLSM) Olympus Fluo View 1000 (Olympus Corporation, Tokyo, Japan). Sample preparation and scanning procedures followed the methods outlined in previous studies (Kuyukina et al., 2014; Ivshina et al., 2019). Image processing and analysis, including the determination of root mean square roughness of the cell surface, cell length, and cell width, were performed using Igor Pro 6.22A software (WaveMetrics, Portland, OR, United States). Furthermore, cell volume and surface area were calculated using the formulas provided in a study (Neumann et al., 2005).

#### 2.2.4 Ultrastructure of bacterial cells

The IEGM 1243 cells grown for 72 h on solid media, including nutrient agar (NA), NA + 50 mg/L IBP, and mineral salt agar (MSA) + 50 mg/L IBP, were harvested, fixed in a solution containing 2.5% glutaraldehyde (w/v) in 0.1 M sodium cacodylate buffer (pH 7.2) for 2.5 h. Subsequently, the fixed cells underwent post-fixation using a 1% (w/v) osmium tetroxide solution in the same buffer. To prepare the samples for microscopy, the fixed cells were dehydrated by a series of ethanol solutions, including absolute ethanol saturated with uranyl acetate, followed by embedding in araldite (Ivshina et al., 2022a). Thin sections were generated using an 8800 Ultrotome III (LKB-Produkter, Stockholm, Sweden), and these sections were stained with lead citrate. The resulting ultrathin sections were examined using a JEM-1400 electron microscope (Japanese Electron Optics Laboratory, Tokyo, Japan).

#### 2.2.5 Elemental mapping of bacterial cells

The harvested IEGM 1243 cell suspensions (as described in section 2.2.4) were directly placed onto copper grids coated with formvar and carbon reinforcement, without the addition of any fixatives. These grids were then allowed to air-dry. For the analysis, transmission electron microscopy combined with energy-dispersive X-ray analysis (TEM-EDX) was performed using the JEM-1400 microscope (Japanese Electron Optics Laboratory, Tokyo, Japan) equipped with an EDXA system (Inca Energy-350, Oxford Instruments, Abingdon, United Kingdom). The microscope operated at an accelerating voltage of 80 keV with a tilt angle of 15° (Ivshina et al., 2022a). The acquisition of EDX spectra and elemental maps was carried out using AZtec software (Oxford Instruments, Abingdon, United Kingdom). In this analysis, only the main Kα peaks of carbon (C), oxygen (O), nitrogen (N), phosphorous (P), potassium (K), sodium (Na), copper (Cu), calcium (Ca), magnesium (Mg), sulfur (S), chlorine (Cl), silicon (Si), and iron (Fe) were taken into consideration.

### 2.3 Catalase activity

The catalase activity of *R. cerastii* IEGM 1243 was determined spectrophotometrically following the protocol described by Gogoleva et al. (2012). Bacterial cells cultured with glucose with and without NSAIDs were centrifuged at 3,000 rpm for 5 min, washed with phosphate buffer (pH 7.0), and resuspended in the same buffer to obtain an optical density (OD_492_) of 0.2. Next, a 0.00125 M hydrogen peroxide (H_2_O_2_) solution (1 mL) was added to 200 μL of the cell suspension, and the mixture was incubated for 10 min at room temperature. To stop the decomposition of H_2_O_2_ by catalase, 100 μL of 2 M HCl solution was added. Subsequently, a 0.025 M potassium iodide solution (1 mL) was added to the resulting mixture, gently mixed, and centrifuged at 3,000 rpm for 15 min. Distilled water was used in control samples instead of cell suspensions. Finally, the absorbance of the supernatant was measured at 492 nm using a Lambda EZ201 spectrophotometer (Perkin-Elmer, Waltham, MA, United States).

The catalase activity was calculated using the equation:


$$A_{\mathrm{cat}}=\frac{12.5\left(1-{OD}_{V}/{OD}_{C}\right)}{T\times {OD}_{b}}$$

where *A*_cat_ represents the catalase activity in μM/min × OD; OD_v_ is the optical density of the supernatant in the experimental sample (in arbitrary units of optical density); OD_c_ is the optical density of the supernatant in the control sample (in arbitrary units of optical density), *T* is the incubation time of bacteria in the presence of H_2_O_2_ (10 min); and OD_b_ is the optical density of the bacterial suspension (OD_492_ 0.2). The catalase activity was expressed as a percentage of the initial level.

### 2.4 Respirometry

Cell respiration activity was evaluated using a 10-channel Micro-Oxymax^®^ respirometer (Columbus Instruments, Columbus, OH, United States) (Ivshina et al., 2019). The experiments were conducted in 300 mL Micro-Oxymax glass flasks containing 100 mL of mineral salt medium with constant agitation (160 rpm, 28 ± 2°C). The rate (μL/h) and accumulation (μL) of oxygen consumption were measured. Automated recording of respiratory activity was performed every 30 min over a period of 7 days.

### 2.5 Analytical procedure

The removal rates of IBP and DCF were determined using high-performance liquid chromatography (HPLC) on an LC Prominence chromatograph (Shimadzu Corporation, Kyoto, Japan) equipped with a reverse-phase C18 column, 25 cm × 4.6 mm, 5 μm (Supelco Inc., Bellefonte, PA, United States) and a diode array detector. Mobile phase consisted of a phosphate buffer solution (pH 5.0) and acetonitrile, mixed in a 60:40 ratio. Elution was performed isocratically at a flow rate of 0.5 mL/min, and detection was carried out at a wavelength of 254 nm. The injection volume was 20 μL, and the column thermostat temperature was set at 40°C. Under these conditions, the retention times for IBP and DCF were determined to be 9.28 ± 0.03 and 18.70 ± 0.02 min, respectively. Prior to analysis, samples were prepared by centrifuging for 5 min at 10,000 rpm. The resulting supernatants were then filtered using a 0.20 μm nylon membrane filter (Nantong FilterBio Membrane Co., Ltd., Nantong, China). The concentrations of DCF and IBP were calculated by comparing the areas under the peaks with those of the standard solutions.

### 2.6 Data analysis and statistics

For cell analysis and quantification, ImageJ software (US National Institutes of Health, Bethesda, Maryland, United States) was employed. A detailed protocol for image processing can be found here (https://www.allevi3d.com/livedead-assay-quantification-fiji/ accessed on 31 May 2023).

Statistical analyses were performed to determine the differences in the experimental data using one-way ANOVA followed by two-sided Dunnett’s t-test. The software used for conducting the statistical tests was SPSS 23.0 (IBM, Armonk, New York, United States). The significance level for the results was set at *p* < 0.05. Each experiment was performed in triplicate to ensure accuracy and reproducibility of the results. For presenting the data from repeated experiments, the values were expressed as mean ± standard deviation.

## 3 Results and discussion

### 3.1 Catalytic activity of *Rhodococcus cerastii* IEGM 1243 towards NSAIDs

**Figure 1:**
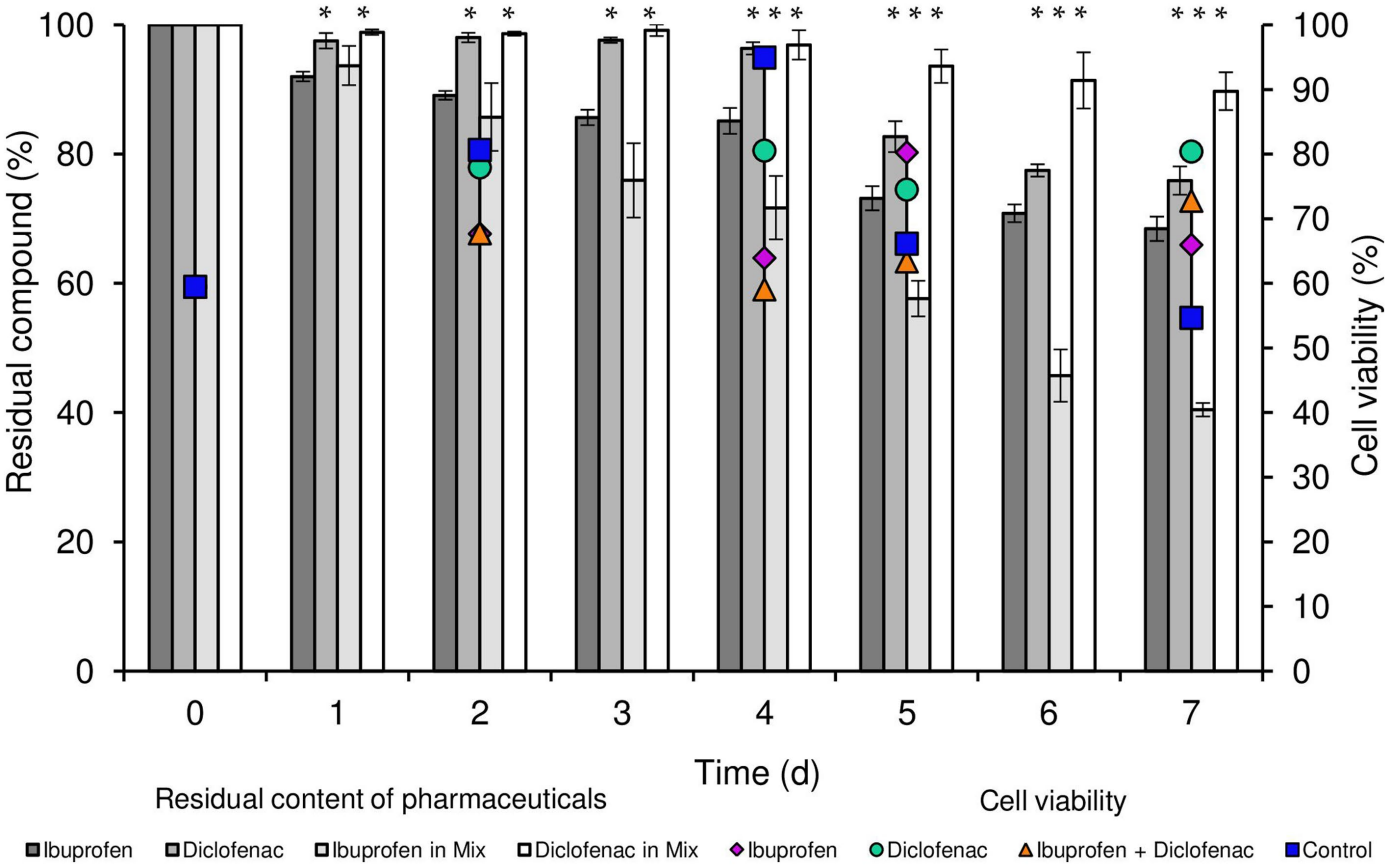
Residual content of NSAIDs and cell viability of *R. cerastii* IEGM 1243 during the biodegradation process. Cells were cultivated in mineral salt medium supplemented with 0.5 g/L glucose (control) and 50 mg/L IBP, 50 mg/L DCF or their mixture (50 mg/L IBP + 50 mg/L DCF). Asterisks indicate significant differences between control and treatments (*p* < 0.05).

According to our data, *R. cerastii* IEGM 1243 exhibited moderate biodegradative activity towards the tested pharmaceutical compounds (Figure 1). When considering individual NSAIDs, the residual content of IBP after a 7 days period was 68.4%, while that of DCF was 75.9%. Interestingly, when using the combination of NSAIDs, the degradation of IBP was higher, with a residual content of 40.4% after 7 days. However, the degradation of DCF in this case was negligible, with a residual content of 89.7%. No losses of substances were observed in abiotic and sorption controls, indicating the biocatalytic nature of the degradation process.

In our previous studies, we confirmed that *R. cerastii* strain IEGM 1278 was capable of completely cometabolizing 100 mg/L IBP within 6 days in the presence of *n*-hexadecane (Ivshina et al., 2021b). Other research showed that IBP could be efficiently degraded at high concentrations (200–1,000 mg/L) by both Gram-negative strains (*Sphingomonas* sp. Ibu-2, *Variovorax* sp. Ibu-1) and Gram-positive strains [*Bacillus thuringiensis* B1(2015b), *Citrobacter freundii* PYI-2, *C. portucalensis* YPI-2, *Nocardia* sp. NRRL 5646, *Patulibacter* sp. I11] (Jan-Roblero and Cruz-Maya, 2023). On the other hand, DCF is more recalcitrant to biodegradation. Previously, we isolated *R. ruber* strain IEGM 346, which was able to degrade only 50% of 50 mg/L DCF within 60 days (Ivshina et al., 2019). In other reports, only Gram-negative *Klebsiella* sp. KSC exhibited the capability to degrade high concentrations of DCF (70 mg/L) within three days, while other strains such as *Pseudomonas moorei* KB4, *Labrys portucalensis* F11, *Raoultella* sp. DD4 degraded much lower concentrations (ranging from 0.5 to 7 mg/L) over up to 28 days (Wojcieszyńska et al., 2023). The results obtained in our study indicate that further optimization of biodegradation conditions for IEGM 1243 is necessary, as our findings do not stand out in this regard.

However, it is important to note that the biodegradation pattern was not the primary focus of our research. We previously outlined the putative biodegradation pathways of DCF and IBP in Ivshina et al. (2019, 2021b). Additionally, Guzik and Wojcieszyńska’s (2019) thorough review covered the microbiological degradation pathways of NSAIDs. Instead, our study primarily concentrated on the physiological and morphological responses of rhodococci when exposed to NSAIDs, which are presented below.

Concurrently with the biodegradation experiments, we conducted quantitative assessments of total cell count and cell viability percentage (Figure 1; Supplementary Figures S1, S2). The initial cell count was determined to be 0.96 cells/mL × 10^7^. Throughout the duration of the experiment, an increase in biomass was consistently observed across all experimental conditions, with the most significant growth recorded in both the control and in the presence of IBP. The highest cell count was attained when IBP was present, peaking at 3.32 cells/mL × 10^7^ after 4 days of the experiment. In parallel, the viability of rhodococci in the control sample exhibited an upward trend until day 4, with viability levels rising from 59.5 to 94.9%, followed by a decline to 54.7% on day 7. In the presence of IBP, the maximum cell viability was reached on day 5 (80.2%), gradually decreasing to 65.9% on day 7. Notably, the overall cell count in the presence of DCF and the NSAID mixture on day 7 of the experiment amounted to 2.1 and 1.59 cells/mL × 10^7^, respectively. Interestingly, these conditions exhibited the highest proportion of viable cells at the end of the experiment.

**Figure 2:**
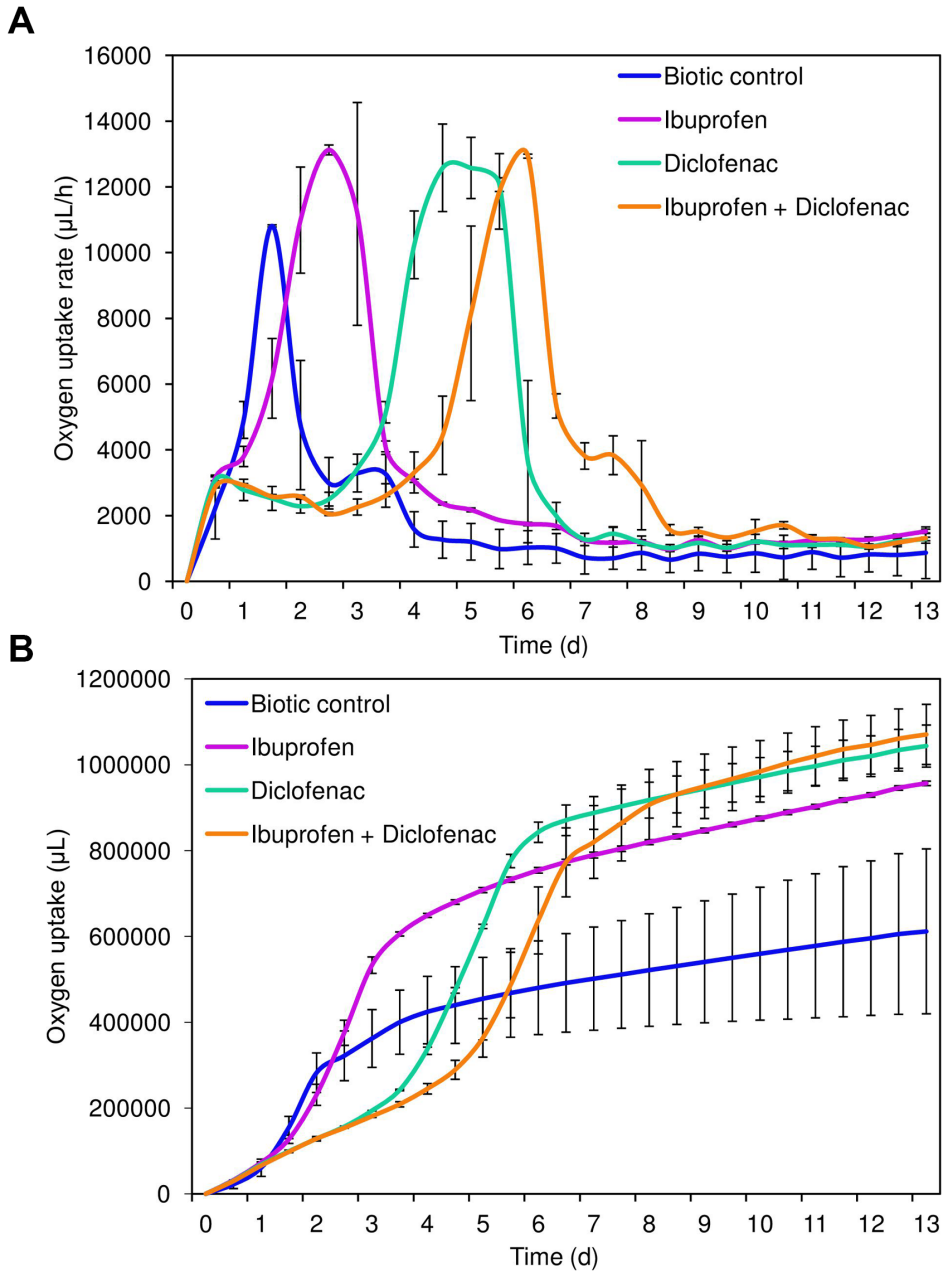
Oxygen uptake rate **(A)** and cumulative oxygen consumption **(B)** by *R. cerastii* IEGM 1243 cells exposed to NSAIDs. Cells were cultivated in mineral salt medium supplemented with 0.5 g/L glucose (control) and 50 mg/L IBP, 50 mg/L DCF or their mixture (50 mg/L IBP + 50 mg/L DCF).

Analysis of cellular respiration in the presence of NSAIDs revealed the following patterns (Figure 2). Both in the biotic control and in the presence of IBP, the lag phase of IEGM 1243 cells was less than 24 h (Figure 2A). The maximum rate of oxygen consumption in the control was reached after 1.5 days of the experiment, amounting to 4753.7 μL/h. In contrast, in the presence of IBP, this parameter was recorded after 2.5 days of the experiment, with a significantly higher value of 13125.4 μL/h.

The transition of rhodococci to the stationary growth phase (Figure 2B) correlated with a slowdown in the biodegradation of NSAIDs (Figure 1). DCF and the mixture exerted a suppressive effect on the metabolic activity of rhodococci for a period of 3–4 days. The maximum rate of oxygen consumption in the presence of DCF was 12580.9 μL/h after 4.5 days of the experiment. Under the combination of IBP and DCF, the peak oxygen consumption was observed on day 6, measuring 12936.4 μL/h.

Cumulative oxygen uptake analysis demonstrated that cells exhibited higher metabolic activity in the presence of NSAIDs (Figure 2B). For instance, on day 9, the total amount of oxygen consumed in the control was 611911.4 μL, whereas in the presence of IBP, DCF, and their mixture, these values were 956805.2 μL, 1,043,623 μL, and 1,070,803 μL, respectively. The increased amount of oxygen consumed in the presence of DCF and the NSAID mixture after 7 days of the experiment correlates with data on higher cell viability under these conditions (Figure 1; Supplementary Figure S2).

Catalase, as a vital antioxidant enzyme, plays a crucial role in the reduction of hydrogen peroxide (H_2_O_2_) to oxygen (O_2_) and water (H_2_O). This enzyme holds significant importance in bacterial stress response and the degradation of xenobiotics (Wang et al., 2000, 2020; Kaushal et al., 2018). *Rhodococcus* spp. are well-known for their high abundance of catalases (Yuan et al., 2021), which makes them robust and adaptable microorganisms. In the context of xenobiotic degradation, including the biodegradation of NSAIDs, the high levels of catalases in rhodococci provide significant benefits. The presence of NSAIDs can lead to the production of harmful reactive oxygen species in the bacteria. However, thanks to their abundant catalases, *Rhodococcus* spp. may efficiently neutralize these ROS, ensuring their metabolic activity and successful degradation of the xenobiotics.

**Figure 3:**
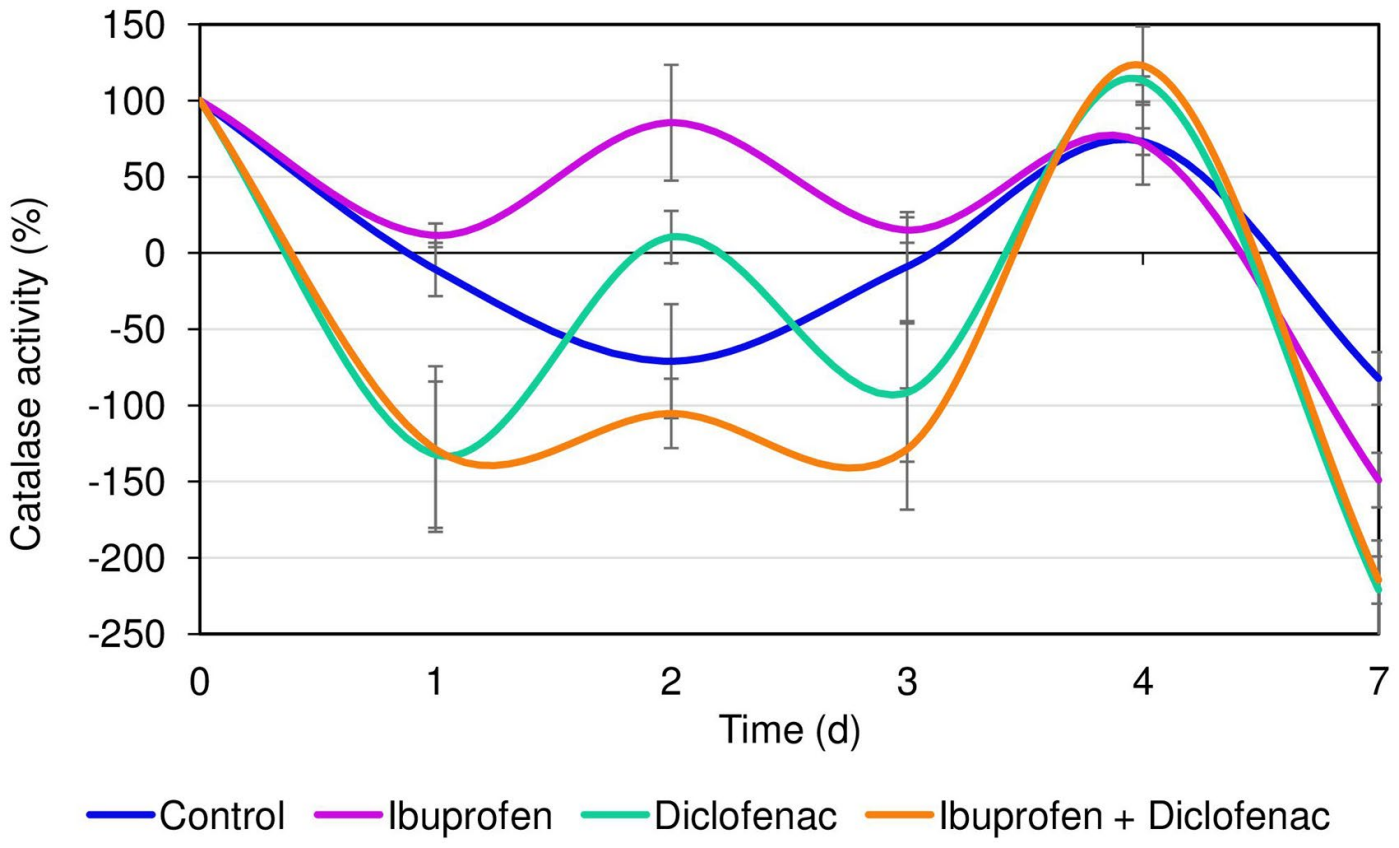
Catalase activity of *R. cerastii* IEGM 1243 in the presence of NSAIDs. Cells were cultivated in mineral salt medium supplemented with 0.5 g/L glucose (control) and 50 mg/L IBP, 50 mg/L DCF or their mixture (50 mg/L IBP + 50 mg/L DCF).

The initial catalase activity of *R. cerastii* IEGM 1243 was 0.66 relative units corresponding to 100%. Within the first day, a decrease in catalase activity was observed in all tested variants. However, the most significant (*p* < 0.05) changes in catalase activity of rhodococci were observed in the presence of DCF and the NSAID mixture (Figure 3). The decrease in catalase activity under these conditions during the first 3 days can be attributed to the cells being in the lag phase of growth (refer to Figure 2A). Interestingly, on the second day, there was an increase in catalase activity in the presence of NSAIDs, while in the control group, the activity continued to decrease. The opposite pattern was observed on the third day. The increase in catalase activity in the samples with NSAIDs on day 4 can be explained by the bacteria entering the exponential growth phase, active degradation of NSAIDs (as seen in Figure 1), and possible accumulation of metabolites. The decrease in catalase activity in all samples on day 7 could be associated with the transition to the stationary growth phase, slowing down of cellular metabolism, and the formation of multicellular aggregates (Supplementary Figure S2) (Wood and Sørensen, 2001). Moreover, it is hypothesized that cell aggregation enhances intercellular synergy, leading to a reduced need for cells to secrete the necessary enzymes (D’Souza et al., 2023). By forming multicellular aggregates, bacterial cells can establish communication networks that facilitate the exchange of genetic material and metabolic by-products. This can lead to a more efficient distribution of tasks among the cells, allowing some individuals to focus on enzyme production while others specialize in different functions. Consequently, this division of labor within the aggregated community can reduce the overall demand for enzyme secretion by individual cells.

### 3.2 Morphometric parameters of *Rhodococcus cerastii* IEGM 1243 cells exposed to NSAIDs

**Figure 4:**
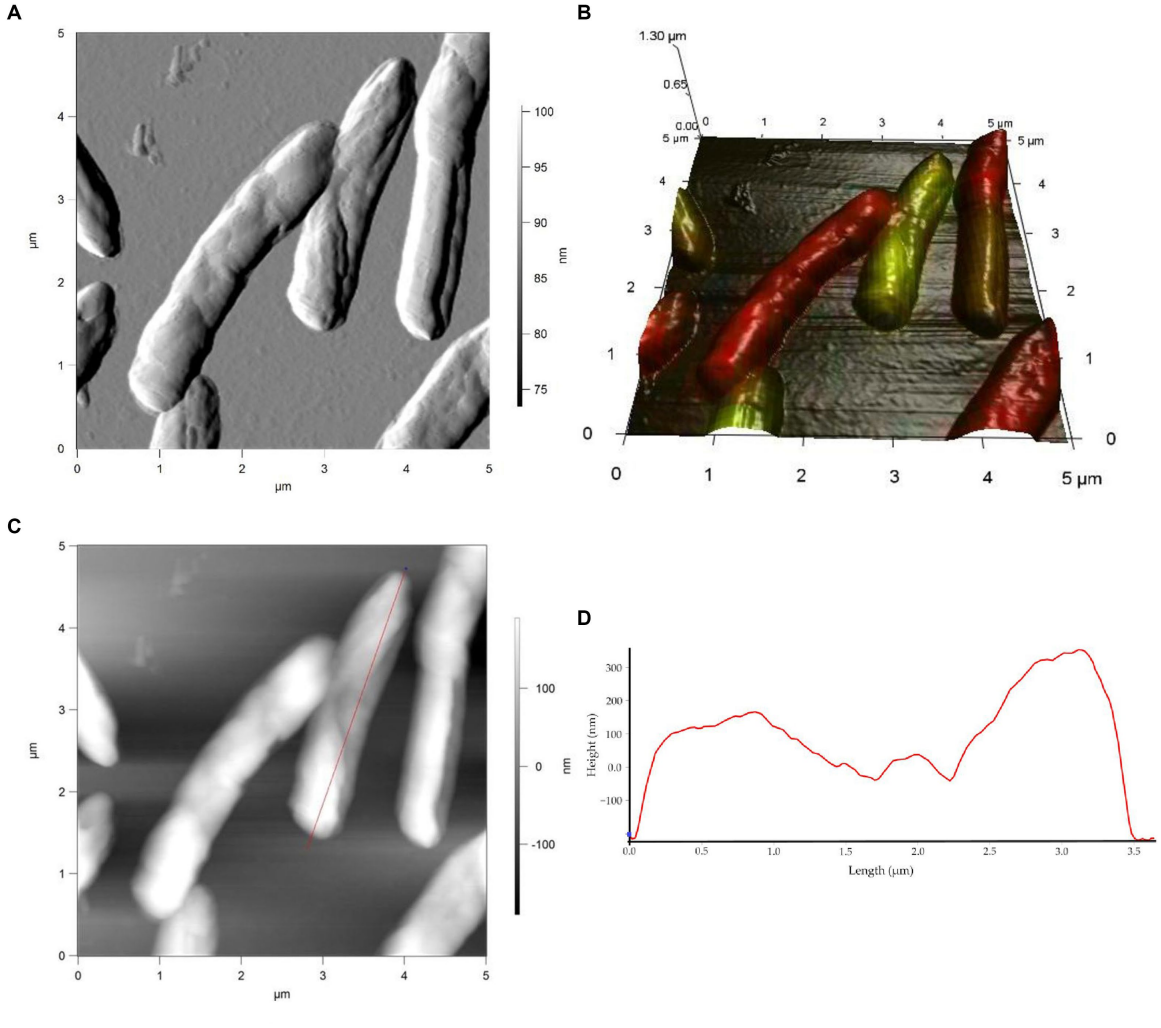
AFM **(A)**, AFM-CLSM **(B)** images, height **(C)** and profile **(D)** of *R. cerastii* IEGM 1243 cells on day 0.

**Figure 5:**
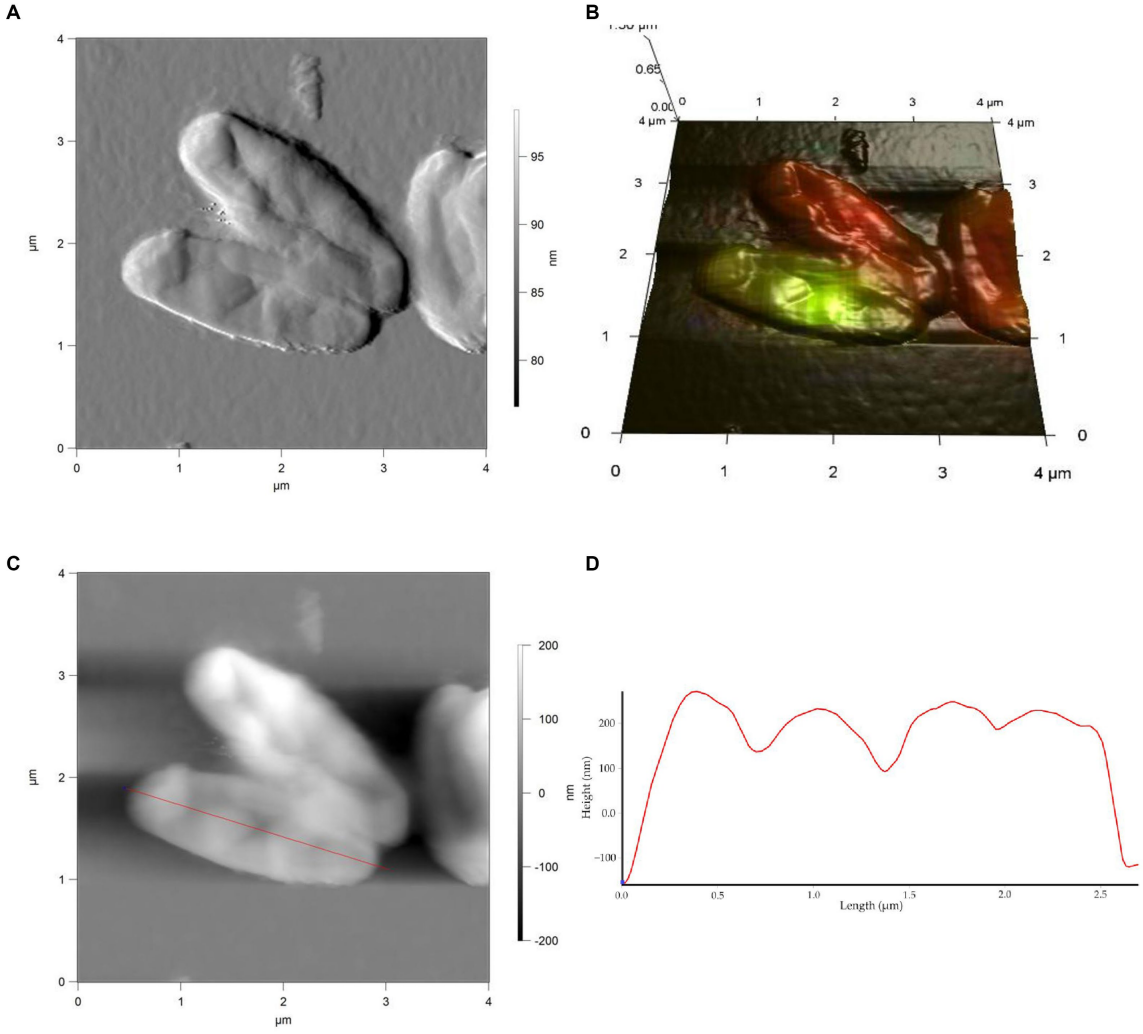
AFM **(A)**, AFM-CLSM **(B)** images, height **(C)** and profile **(D)** of *R. cerastii* IEGM 1243 cells grown in mineral salt medium supplemented with 0.5 g/L glucose for 4 days.

**Figure 6:**
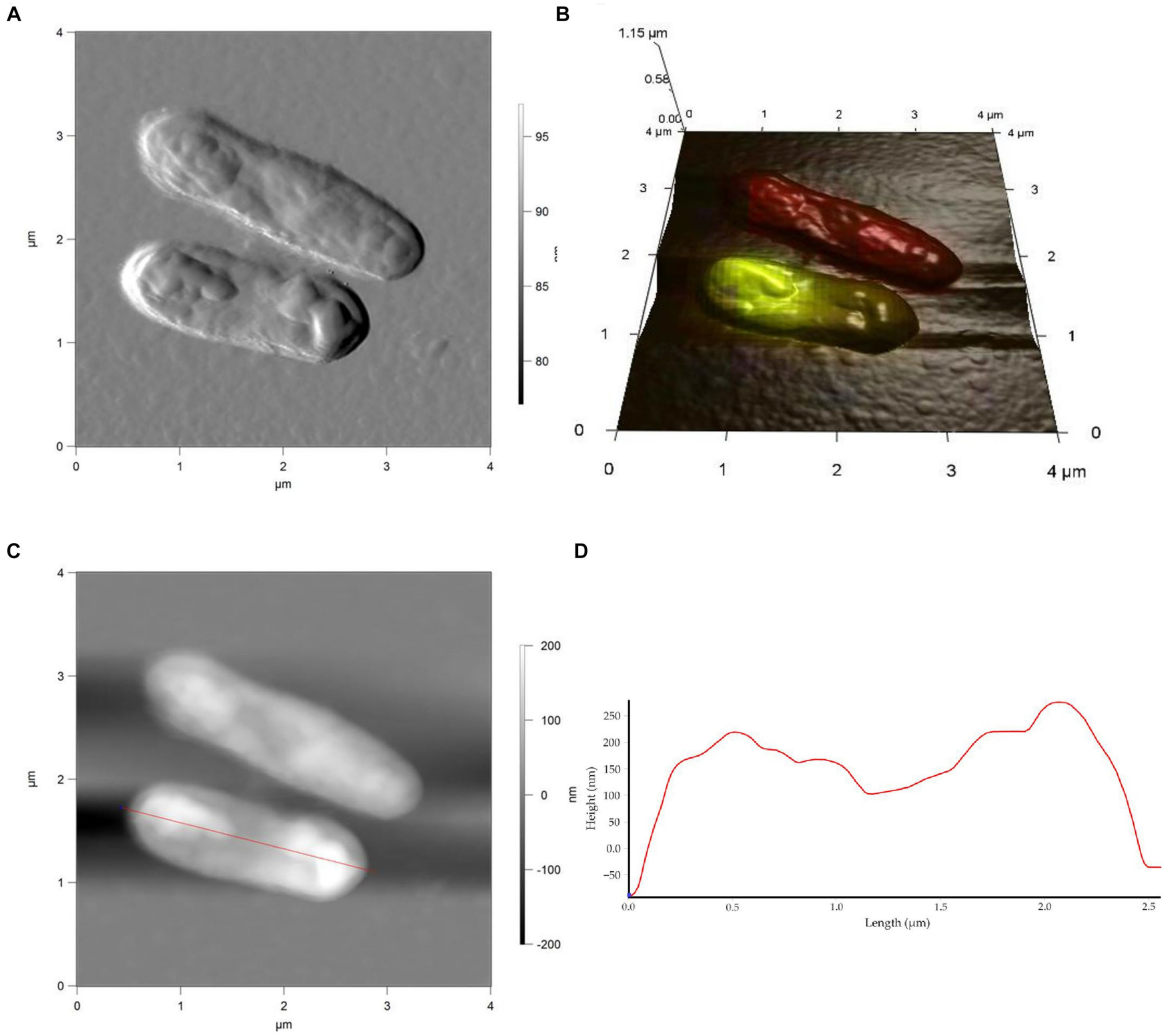
AFM **(A)**, AFM-CLSM **(B)** images, height **(C)** and profile **(D)** of *R. cerastii* IEGM 1243 cells grown in mineral salt medium supplemented with 0.5 g/L glucose and 50 mg/L IBP for 4 days.

**Figure 7:**
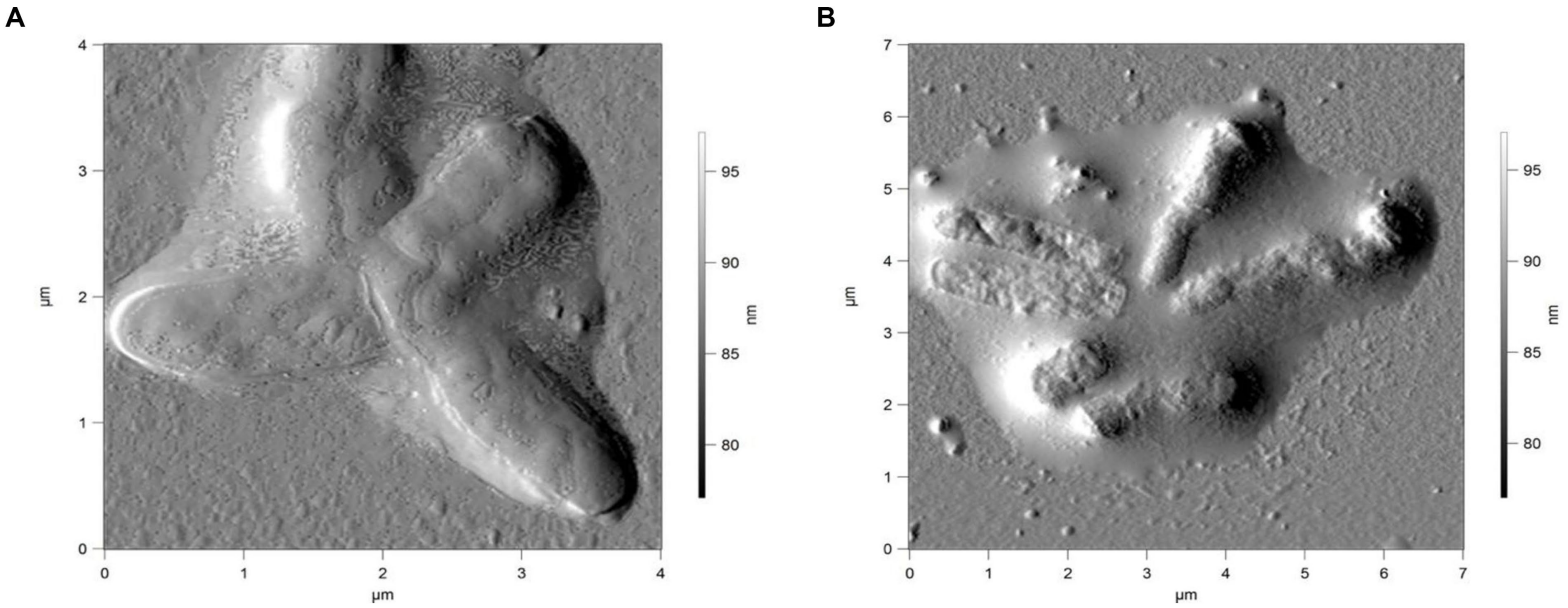
AFM of *R. cerastii* IEGM 1243 cells grown in mineral salt medium supplemented with 0.5 g/L glucose and 50 mg/L DCF **(A)** or 50 mg/L IBP + 50 mg/L DCF **(B)** for 4 days.

Studies on cell morphology and morphogenesis are of great importance for understanding cell growth, reproduction, and adaptation to the environment (Young, 2006; Levin and Angert, 2015; Ojkic et al., 2019; Shi et al., 2021; Govers et al., 2023). In this study, we analyzed the morphometric parameters of live cells of *R. cerastii* IEGM 1243 exposed to NSAIDs, and the results are presented in Figures 4–7, Table 1, and Supplementary Figures S3–S5. Initially, in their native state, the *R. cerastii* IEGM 1243 cells exhibited an elongated rod-shaped morphology (Figures 4A,B) with distinct peaks and valleys (Figure 4D), as well as a rough cell surface.

**Table 1:** Morphometric features of *R. cerastii* IEGM 1243 cells grown in mineral salt medium supplemented 0.5 g/L with glucose (control) and 50 mg/L NSAIDs.

Treatment	Length, μm	Width, μm	Surface area (SA), μm^2^	Volume (V), μm^3^	SA/V, μm^−1^	Root-mean-square roughness, nm
**Day 0**						
Control	2.91 ± 0.44	0.76 ± 0.10	7.89 ± 1.87	1.35 ± 0.50	6.08 ± 0.80	47.71 ± 15.90
**Day 4**						
Control	2.56 ± 0.53	0.80 ± 0.14	7.52 ± 2.32	1.35 ± 0.65	5.96 ± 0.91	65.15 ± 20.17
IBP	2.85 ± 0.68*	0.82 ± 0.09	8.40 ± 2.04*	1.52 ± 0.52	5.67 ± 0.57	118.03 ± 46.58*
DCF	2.64 ± 0.76	0.75 ± 0.08*	7.06 ± 2.00	1.16 ± 0.42	6.24 ± 0.60	nd
DCF + IBP	2.67 ± 0.69	0.79 ± 0.12	7.66 ± 2.30	1.35 ± 0.60	5.97 ± 0.84	nd
**Day 7**						
Control	2.56 ± 0.56	1.00 ± 0.21	9.65 ± 2.71	2.08 ± 0.97	4.96 ± 0.81	113.55 ± 36.62
IBP	2.54 ± 0.54	0.78 ± 0.14**	7.26 ± 2.23**	1.27 ± 0.68**	6.05 ± 0.78**	130.35 ± 35.12
DCF	2.59 ± 0.67	0.79 ± 0.12**	7.40 ± 2.19**	1.29 ± 0.58**	6.01 ± 0.75**	235.46 ± 72.87**
DCF + IBP	2.85 ± 0.72**	0.81 ± 0.12**	8.28 ± 2.19**	1.49 ± 0.56**	5.81 ± 0.81**	219.34 ± 39.72**

Values significantly (*p* < 0.05) differ from controls on the fourth day (*), and on the seventh (**) day. The results are presented as mean ± standard deviation (*n* = 100).

Rod-shaped bacteria have the ability to readily adapt their shape in response to changes in the surrounding environment (Chang and Huang, 2014; Ojkic et al., 2019). In our study, we observed that the control cells underwent shape alterations on day 4, with a reduction in length and an increase in width (Figure 5 and Table 1). However, according to height measurements, no significant differences in cell sizes were observed between the NSAID-treated cells and the control (Figures 6, 7 and Table 1). Notably, when exposed to DCF and the NSAID mixture, cells produced a significant amount of extracellular polymeric substances, which hindered the assessment of cell profile and height (Figure 7).

On the seventh day, significant differences in morphometric parameters of cells in the presence of NSAIDs were observed, particularly a decrease in surface area, volume, and an increase in the surface area-to-volume ratio (SA/V) (Table 1). SA/V is an important physical parameter that regulates the influx and efflux of substances and metabolites (Ojkic et al., 2022). The cell size, shape, and SA/V are interconnected, and individual cells can change their size and shape to achieve an optimal SA/V (Harris and Theriot, 2018; Shi et al., 2021). Previously, we observed alterations in the SA/V ratio in rhodococci exposed to pharmaceuticals (Ivshina et al., 2019; Tyumina et al., 2019; Ivshina et al., 2021b, 2022b). For example, when exposed to toxic substrates such as DCF or naproxen, cells tend to reduce their SA/V to minimize the exposed cell surface for contact with the stressor (Ivshina et al., 2019, 2022b). Studies by other authors have shown that in the presence of ampicillin, *Escherichia coli* cells maintain an ellipsoidal shape and a low SA/V to reduce their metabolic activity, conserve energy, and prevent division (Uzoechi and Abu-Lail, 2019). The reduction in metabolic activity, in turn, leads to cell persistence and resistance (Ojkic et al., 2022). On the other hand, in the presence of less toxic compounds or under carbon source limitation, SA/V ratio may increase for more efficient contact between cells and a substrate (Ivshina et al., 2019, 2021b). In the short term, a cell is capable of regulating its shape by adjusting turgor pressure. However, over longer time scales, changes in cell size are predominantly determined by alterations in cell width (Oldewurtel et al., 2021). In our case, on day 7, the NSAID-treated cells reduced their width (*p* < 0.05), which ultimately resulted in smaller cell volume and larger SA/V. Possible reasons for the change in SA/V ratio include the availability of precursors for peptidoglycan biosynthesis and the presence of inhibitors of fatty acid biosynthesis (Harris and Theriot, 2018). Additionally, cell size is influenced by the levels of transport proteins, metabolic activity, and the accumulation of cell division protein (FtsZ) (Ojkic et al., 2019; Bertaux et al., 2020).

### 3.3 Ultrastructural analysis of *Rhodococcus cerastii* IEGM 1243 exposed to ibuprofen

**Figure 8:**
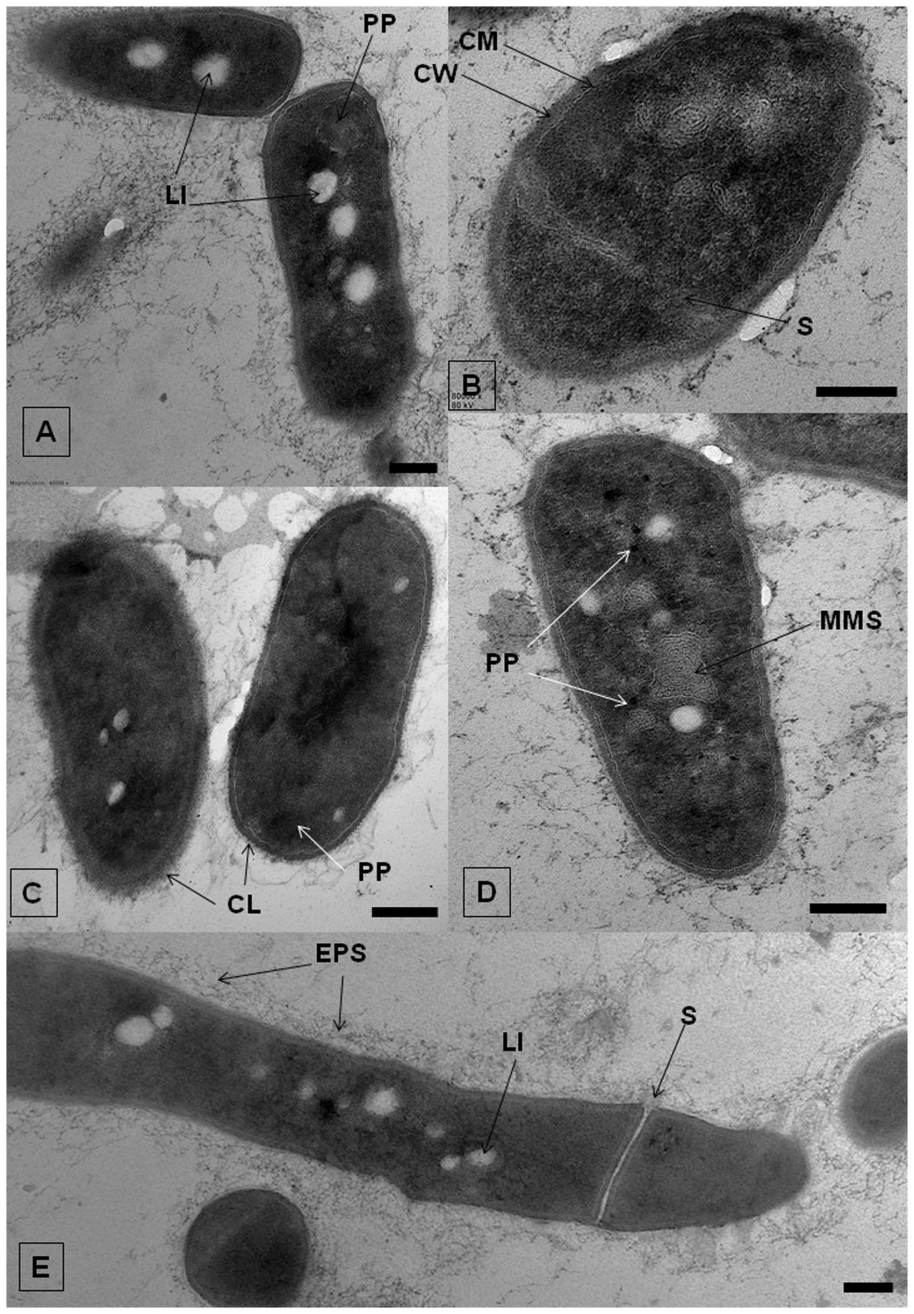
TEM-images of *R. cerastii* IEGM 1243 grown on nutrient agar (control, **A,E**) supplemented with 50 mg/L IBP **(B,D)** or on minimal salt agar supplemented with 50 mg/L IBP **(C)**. Cells were cultivated for 3 days. CL, capsular layer; CM, cytoplasmatic membrane; CW, cell wall; EPS, extracellular polymeric substances; LI, lipid inclusions; MLS, membrane-like structures; PP, polyphosphate granules; S, septum. Scale bars correspond to 200 nm.

The effect of IBP on the ultrastructure of *R. cerastii* IEGM 1243 cells was investigated using transmission electron microscopy (TEM) analysis. TEM provides high-resolution imaging of cellular structures, allowing for detailed observations of cellular ultrastructure. Comparative analysis of ultrathin sections revealed that the morphological traits of rhodococci were similar in cultures developed under both control and IBP-containing conditions (Figure 8; Supplementary Figures S6–S8). As evident from the Figure 8E, strain IEGM 1243 exhibits an apical mode of cell elongation, resulting in the increased accumulation of new cell wall material at the cell poles (Donovan and Bramkamp, 2014). The cells exhibited characteristic features, including the presence of an outer capsular layer, which was visually discernible as protrusions. Furthermore, they displayed a stratified cell wall with an outer layer that appeared electron-dense, indicating the existence of a well-defined cell envelope. Additionally, the cytoplasmic membrane remained intact, indicating the overall structural integrity of the cells.

In both the control and IBP-treated cells, TEM revealed the presence of two distinct types of structures: electron-transparent bodies, likely lipid inclusions, and electron-dense bodies, believed to be polyphosphates. Intracellular lipid inclusions important energy storage compounds that fulfill carbon requirements and maintain redox homeostasis, enabling bacteria to survive for extended periods (Alvarez et al., 1996, 2000; Mallick et al., 2021). Furthermore, they play a crucial role in the pathogenesis of mycobacteria (Mallick et al., 2021). *Rhodococcus* spp. commonly utilize polyhydroxyalkanoates, triacylglycerols, and glycogen as intracellular carbon reserves (Hernández et al., 2008; Cappelletti et al., 2020).

**Figure 9:**
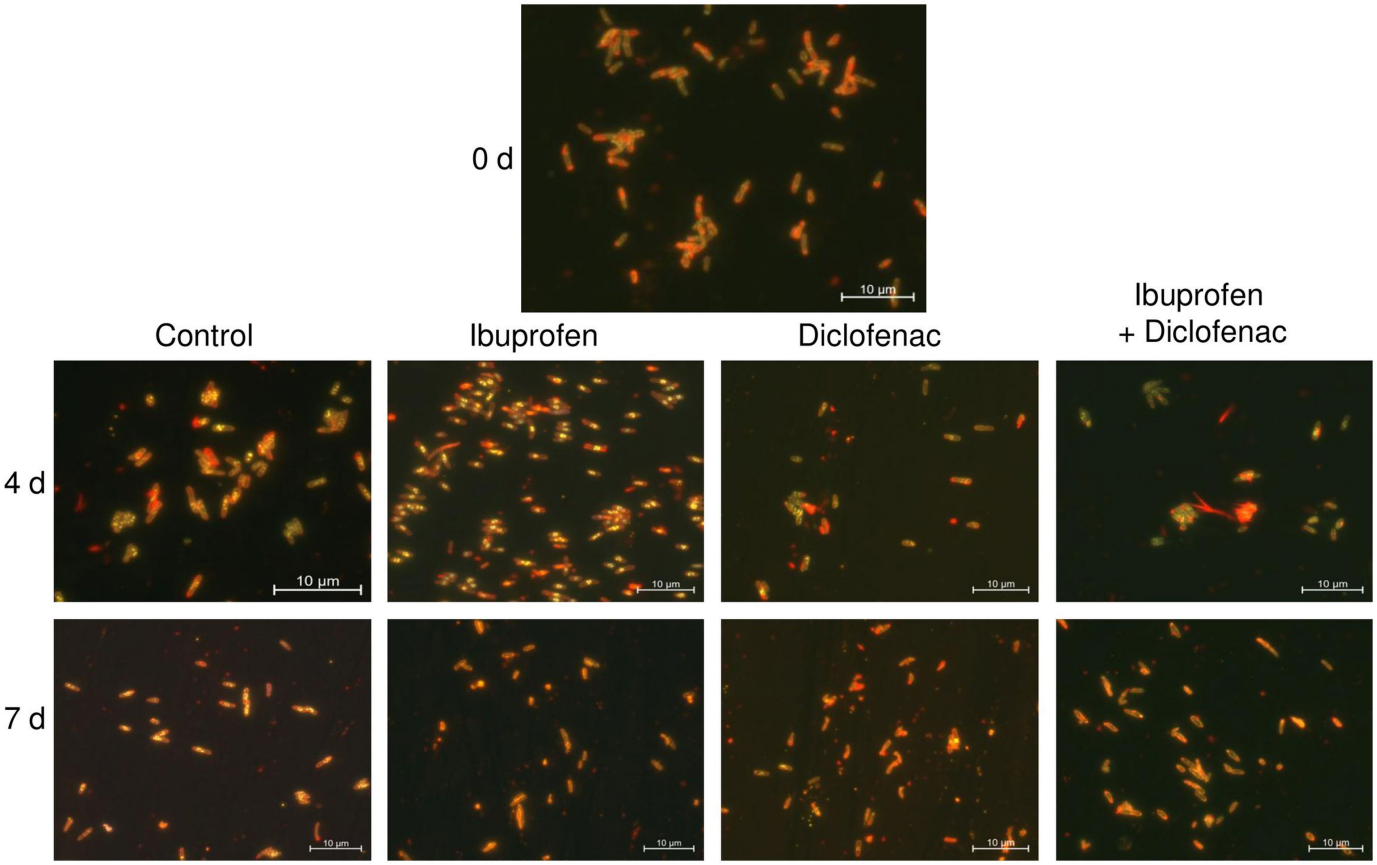
Fluorescent microscopy images of *R. cerastii* IEGM 1243. Cells were cultivated in mineral salt medium supplemented with 0.5 g/L glucose (control) and 50 mg/L IBP, 50 mg/L DCF or their mixture (50 mg/L IBP + 50 mg/L DCF). Yellow dots are lipid inclusions.

Nile Red staining of the bacteria revealed the presence of a small amount of lipid inclusions, mainly located at the periphery of the cells, at the initial time point (Figure 9). By day 4 of the experiment, there was a tendency for the accumulation of lipid inclusions in all treatments, particularly evident in the control and in the presence of IBP. The number of inclusions reached up to 10 per cell, predominantly localized in the central part of the cell. In the case of DCF and the NSAID mixture, the quantity and size of lipids were smaller, and the inclusions were often located at the periphery of the cells. After 7 days of observation, the control cells maintained a significant amount of lipid inclusions; however, individual cells without intracellular lipids were also observed. When exposed to NSAIDs, cells primarily depleted their lipid reserves, with individual lipid granules located at the periphery of the cells (IBP and NSAID-mixture) or at the center of the cells (DCF). The red fluorescence around the cell perimeter could be associated with the lipids of the cell membranes (Presentato et al., 2018).

According to a previous study (Wältermann et al., 2005), the process of lipid-body synthesis involves the formation of small lipid droplets that remain attached to membrane-associated enzymes. Over time, these droplets aggregate and merge, leading to the formation of larger structures known as membrane-bound lipid prebodies. Eventually, these lipid prebodies are released into the cytoplasm, becoming mature entities within the cell. Our current study corroborates these findings (Supplementary Figure S9). For instance, at the beginning of the experiment, IEGM 1243 cells grown in the presence of glucose (control) exhibited small inclusions at the periphery, which likely represent small lipid droplets. As time progressed, these droplets transitioned into the cytoplasm, forming larger lipid prebodies in the early stages and eventually maturing into large lipid inclusions on days 2 and 3. The formation of lipid inclusions in the presence of IBP followed a similar scenario, while under the influence of DCF and the NSAID mixture, the maturation of lipid inclusions occurred on day 4.

When IBP was present, cells exhibited the formation of intracellular membrane-like structures (Figure 8D; Supplementary Figure S7). These structures appeared as loop-like formations and are believed to be primarily involved in the transportation of complex compounds and their subsequent degradation through the action of membrane-bound enzymes (Royes et al., 2020; Thi Mo et al., 2022). Similar structures have been observed in previous studies involving rhodococci grown in the presence of benzoate (Solyanikova et al., 2017), oleanolic acid (Luchnikova et al., 2022), as well as liquid and solid n-hexadecane (Thi Mo et al., 2022). This suggests a common mechanism or adaptive response in the formation of these membrane-like structures across different environmental conditions and compound exposures.

**Figure 10:**
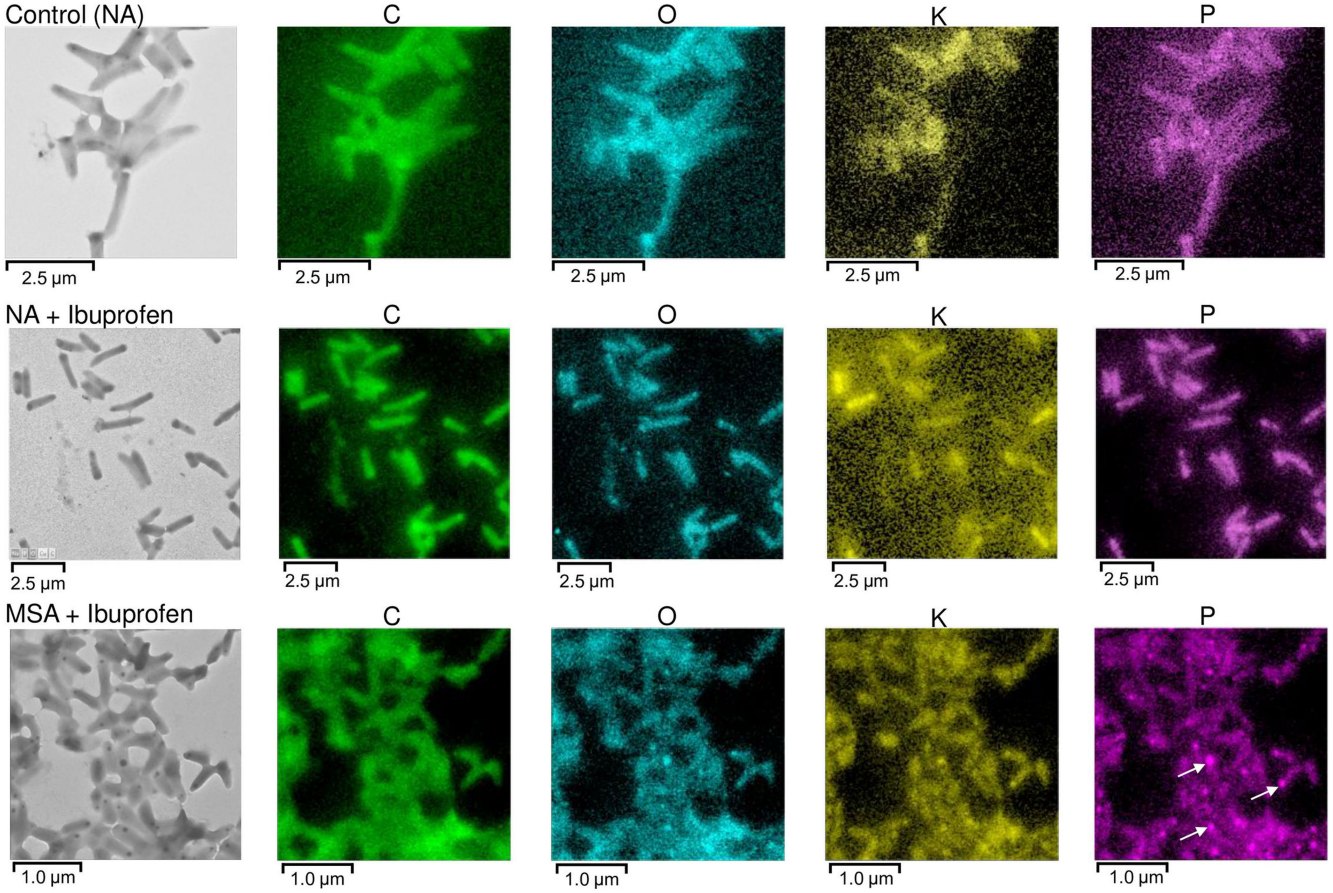
TEM-EDX analysis: Images and elemental mapping (C, O, K, P) of *R. cerastii* IEGM 1243 cells. Cells were grown on nutrient agar (Сontrol, NA) supplemented with IBP (NA + Ibuprofen) or on minimal salt agar supplemented with IBP (MSA + Ibuprofen) for 3 days. Other elements are presented in Supplementary Figures S11–S13. The white arrows indicate polyphosphate inclusions.

Polyphosphates were represented as compartments mainly located at the cell poles (Figures 8A,C). Similar observations of polyphosphates were made in cells of *R. rhodochrous* IEGM 1362 grown in a nutrient-rich medium and in the presence of (−)-isopulegol (Ivshina et al., 2022a), *R. rhodochrous* IEGM 757 in the presence of oleanolic acid (Luchnikova et al., 2022), and *R. erythropolis* N9T-4 in a basal medium without any additional carbon, nitrogen, sulfur, and energy sources (Yoshida et al., 2017). In the latter case, the authors referred to these compartments as oligobodies and confirmed their high phosphorus and potassium content using X-ray spectroscopy. Phosphorus-rich granules are composed of linear chains of polyphosphate and cations such as magnesium, potassium, and calcium (Keim et al., 2005). Polyphosphate inclusions serve as an internal phosphate reserve in cells and play an important role in the adaptive mechanisms of bacteria under suboptimal environmental conditions. In our case, particularly significant accumulations of phosphorus were observed in cells grown on mineral salt agar supplemented with IBP. TEM-EDX analysis showed distinct zones of increased phosphorus accumulation at the cell poles, along with significant amounts of potassium and magnesium (Figure 10; Supplementary Figures S10–S13). Furthermore, the elevated potassium content in the external environment in the presence of IBP may indicate its efflux from the cells due to the disruption of cell membrane permeability (Luchnikova et al., 2022). However, it is essential to note that further investigations are needed to fully understand the underlying mechanisms behind these observations.

## 4 Conclusion and future perspectives

In this work, we investigated how individual and combined NSAIDs affect *Rhodococcus cerastii* strain IEGM 1243. Among the key findings, we observed significant alterations in catalase activity and a noticeable depletion of lipid inclusions in bacterial cells exposed to IBP, DCF, and their combination. These changes in catalase activity are indicative of adjustments in the stress response mechanisms employed by *R. cerastii* strain IEGM 1243 when confronted with NSAID-induced stressors. Furthermore, the observed depletion of lipid inclusions suggests potential modifications in the lipid metabolism pathways, which may play a pivotal role in adapting to NSAID exposure.

Morphometric analysis has revealed significant differences in cell size and surface area-to-volume ratio in the presence of NSAIDs. These alterations in cellular morphology might be influenced by factors related to peptidoglycan biosynthesis and fatty acid biosynthesis inhibitors.

For the first time, we employed high-precision electron microscopy to uncover ultrastructural changes and element mapping of *R. cerastii* IEGM 1243 cells treated with IBP. This advanced technique revealed the structural integrity of the cells, even in the presence of the pharmaceutical. The identification of lipid inclusions, polyphosphates, and intracellular membrane-like structures in IBP-treated cells provides a deeper understanding of the cellular adaptations that occur in response to NSAIDs. These ultrastructural insights open new avenues for research into the mechanisms of bacterial resistance and adaptation at the nanoscale level.

Looking ahead, future prospects in this field should incorporate advanced omics techniques to unravel the underlying genetic, transcriptomic, proteomic, and metabolomic changes in *R. cerastii* IEGM 1243 under NSAID exposure. Such sophisticated analyses can provide a holistic understanding of the bacterial response, shedding light on the intricate molecular mechanisms driving adaptation to pharmaceutical pollution. Additionally, the knowledge gained from understanding the adaptive reactions of bacteria towards pharmaceuticals can be harnessed in the development of biocatalysts for pharmaceutical waste disposal, aligning with the growing need for sustainable and environmentally friendly approaches to address pharmaceutical contamination.

## Data availability statement

The raw data supporting the conclusions of this article will be made available by the authors, without undue reservation.

## Author contributions

ET: Investigation, Visualization, Writing – original draft. GB: Investigation, Writing – original draft. NK: Investigation, Visualization, Writing – review & editing. VS: Investigation, Writing – review & editing. AM: Methodology, Visualization, Writing – review & editing. II: Conceptualization, Data curation, Funding acquisition, Methodology, Project administration, Resources, Supervision, Writing – review & editing.
