# Supplementary material for: Phenotypic and metabolic adaptations of Rhodococcus cerastii strain IEGM 1243 to separate and combined effects of diclofenac and ibuprofen
Source: Front Microbiol. 2023 Dec 6;14:1275553. doi: 10.3389/fmicb.2023.1275553 (PMC10730942; doi:10.3389/fmicb.2023.1275553)
Supplement: Supplementary file 2 [file Image_2.PDF]

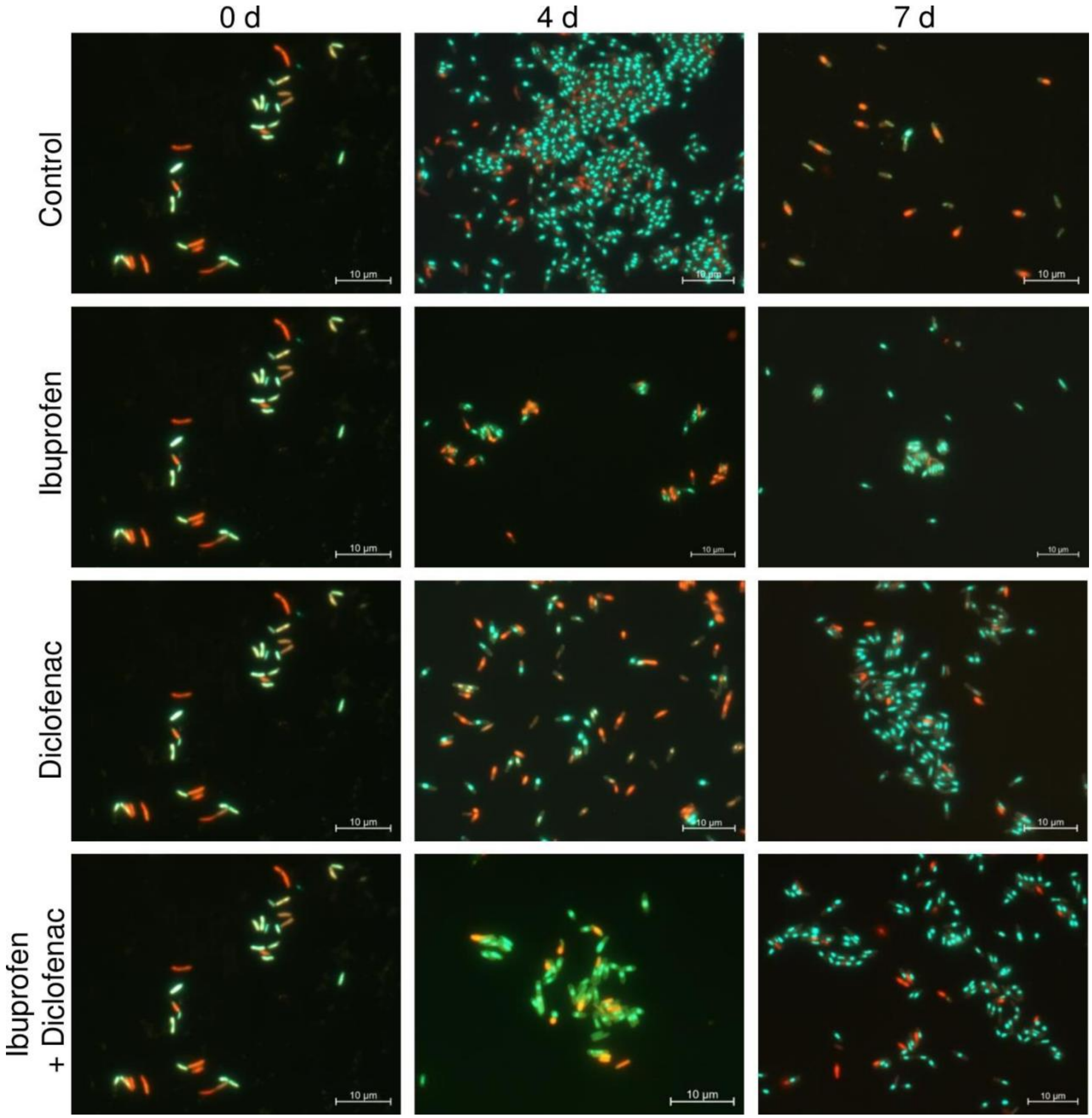

Supplementary Figure 2. Fluorescent microscopy images of *R. cerastii* IEGM 1243. Cells were cultivated in mineral salt medium supplemented with 0.5 g/L glucose (control) and 50 mg/L IBP, 50 mg/L DCF or their mixture (50 mg/L IBP + 50 mg/L DCF). Green/red fluorescent cells are alive/dead.
