# Supplementary material for: Phenotypic and metabolic adaptations of Rhodococcus cerastii strain IEGM 1243 to separate and combined effects of diclofenac and ibuprofen
Source: Front Microbiol. 2023 Dec 6;14:1275553. doi: 10.3389/fmicb.2023.1275553 (PMC10730942; doi:10.3389/fmicb.2023.1275553)
Supplement: Supplementary file 3 [file Image_3.PDF]

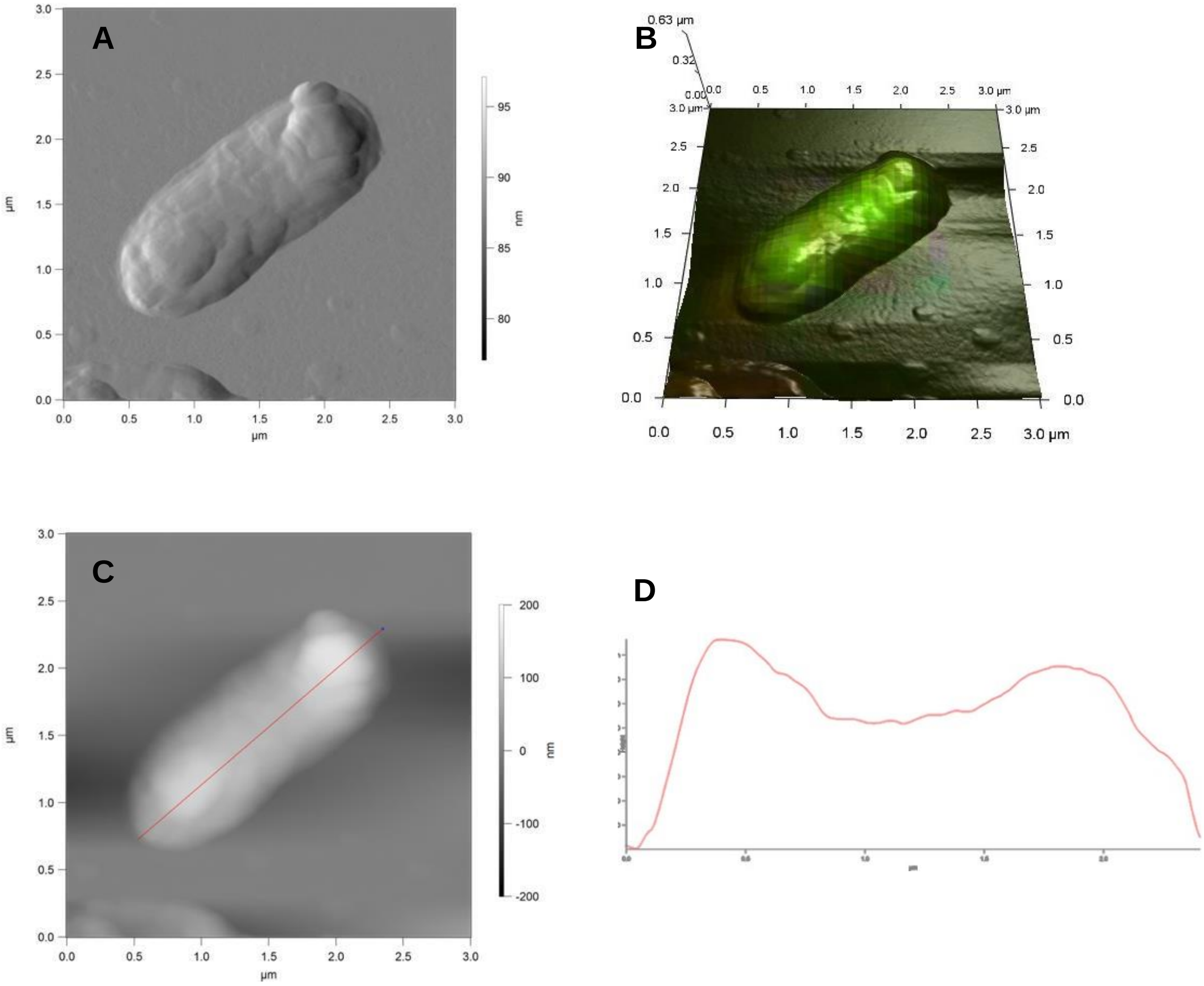

Supplementary Figure 3. AFM (**A**), AFM-CLSM (**B**) images, height (**C**) and profile (**D**) of *R. cerastii* IEGM 1243 cells grown in mineral salt medium supplemented with 0.5 g/L glucose and 50 mg/L IBP for 7 days.
