# Supplementary material for: Phenotypic and metabolic adaptations of Rhodococcus cerastii strain IEGM 1243 to separate and combined effects of diclofenac and ibuprofen
Source: Front Microbiol. 2023 Dec 6;14:1275553. doi: 10.3389/fmicb.2023.1275553 (PMC10730942; doi:10.3389/fmicb.2023.1275553)
Supplement: Supplementary file 8 [file Image_8.PDF]

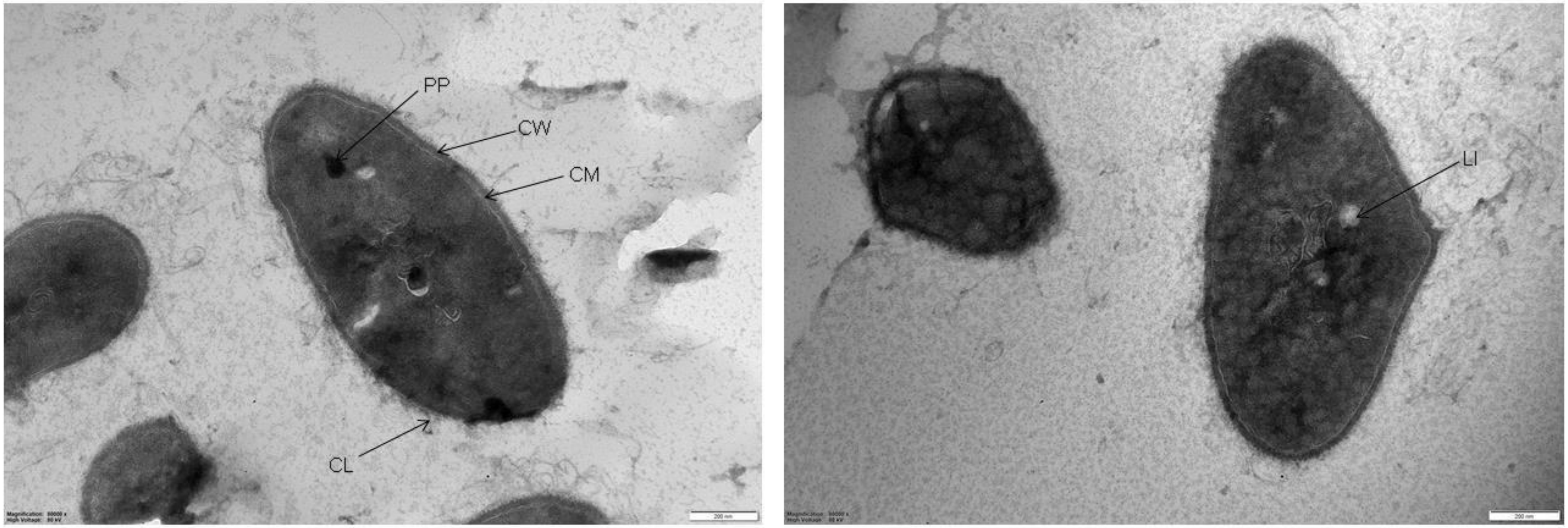

Supplementary Figure 8. TEM-images of *R. cerastii* IEGM 1243 grown on mineral salt agar with 50 mg/L IBP for 3 days. CL – capsular layer, CM – cytoplasmatic membrane, CW – cell wall, EPS – extracellular polymeric substances, LI – lipid inclusions, MLS – membrane-like structures, PP – polyphosphate granules, S – septum. Scale bars correspond to 200 nm.
