# Supplementary material for: Phenotypic and metabolic adaptations of Rhodococcus cerastii strain IEGM 1243 to separate and combined effects of diclofenac and ibuprofen
Source: Front Microbiol. 2023 Dec 6;14:1275553. doi: 10.3389/fmicb.2023.1275553 (PMC10730942; doi:10.3389/fmicb.2023.1275553)
Supplement: Supplementary file 9 [file Image_9.PDF]

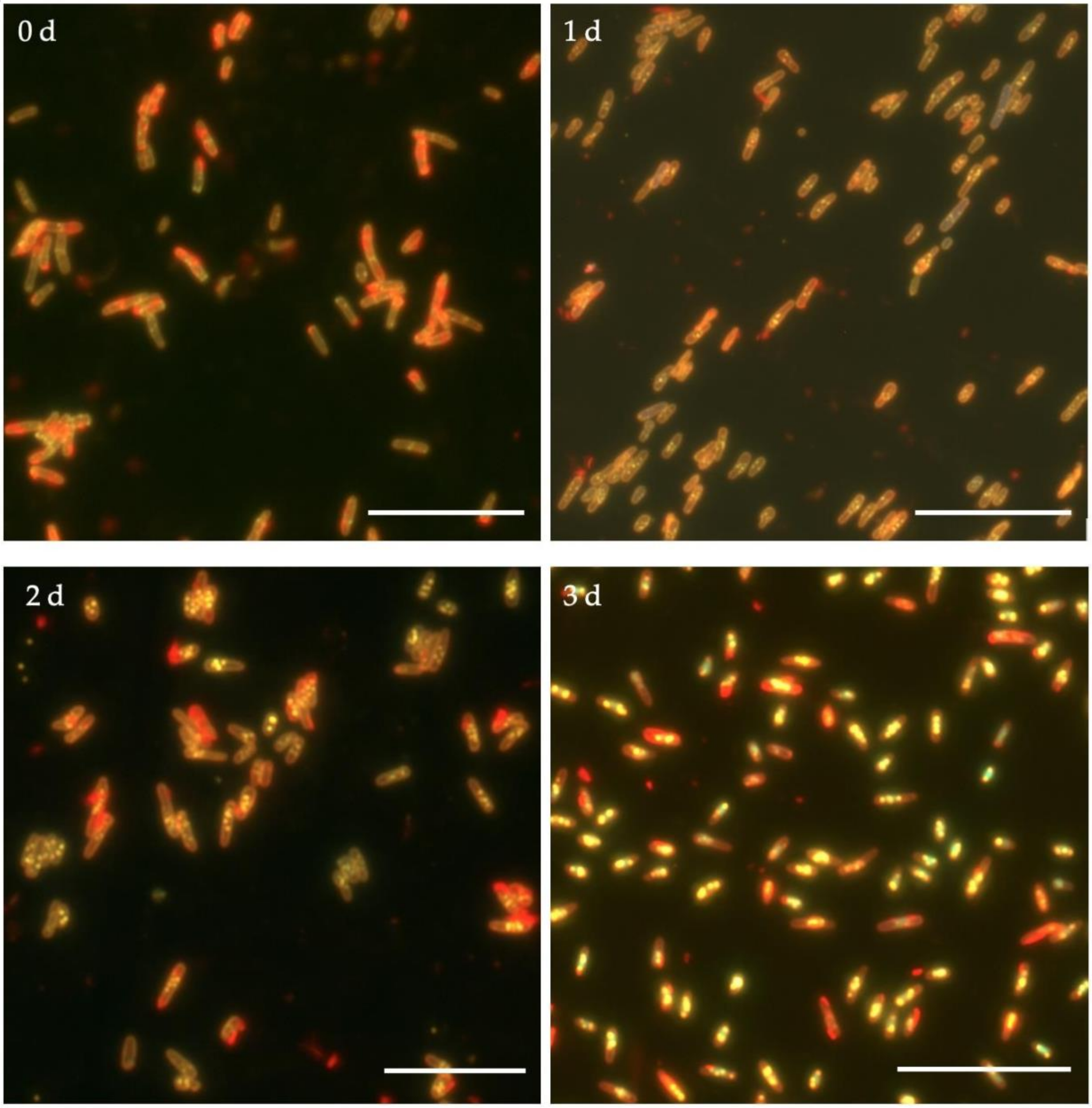

Supplementary Figure 9. Fluorescent microscopy images of *R. cerastii* IEGM 1243. Cells were cultivated in the mineral salt medium supplemented with 0.5 g/L glucose. Scale bars represent 10 µm.
