# Supplementary material for: Phenotypic and metabolic adaptations of Rhodococcus cerastii strain IEGM 1243 to separate and combined effects of diclofenac and ibuprofen
Source: Front Microbiol. 2023 Dec 6;14:1275553. doi: 10.3389/fmicb.2023.1275553 (PMC10730942; doi:10.3389/fmicb.2023.1275553)
Supplement: Supplementary file 10 [file Image_10.PDF]

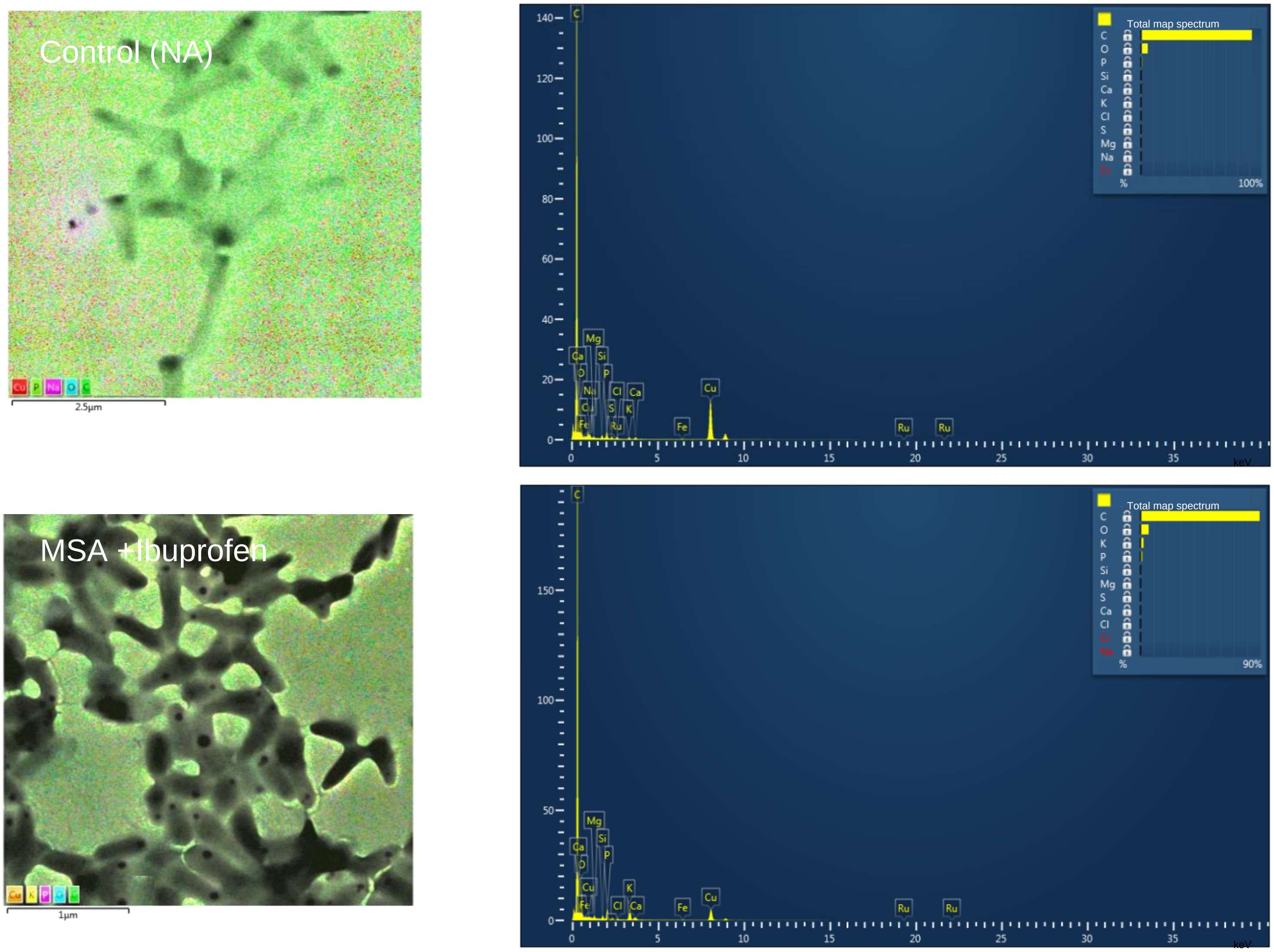

Supplementary Figure. EDX-spectra for *R. cerastii* IEGM 1243 cells. Cells were grown on nutrient agar (NA, control) or on minimal salt agar supplemented with 50 mg/L IBP (MSA + Ibuprofen) for 3 days.
