# Supplementary material for: Phenotypic and metabolic adaptations of Rhodococcus cerastii strain IEGM 1243 to separate and combined effects of diclofenac and ibuprofen
Source: Front Microbiol. 2023 Dec 6;14:1275553. doi: 10.3389/fmicb.2023.1275553 (PMC10730942; doi:10.3389/fmicb.2023.1275553)
Supplement: Supplementary file 13 [file Image_13.PDF]

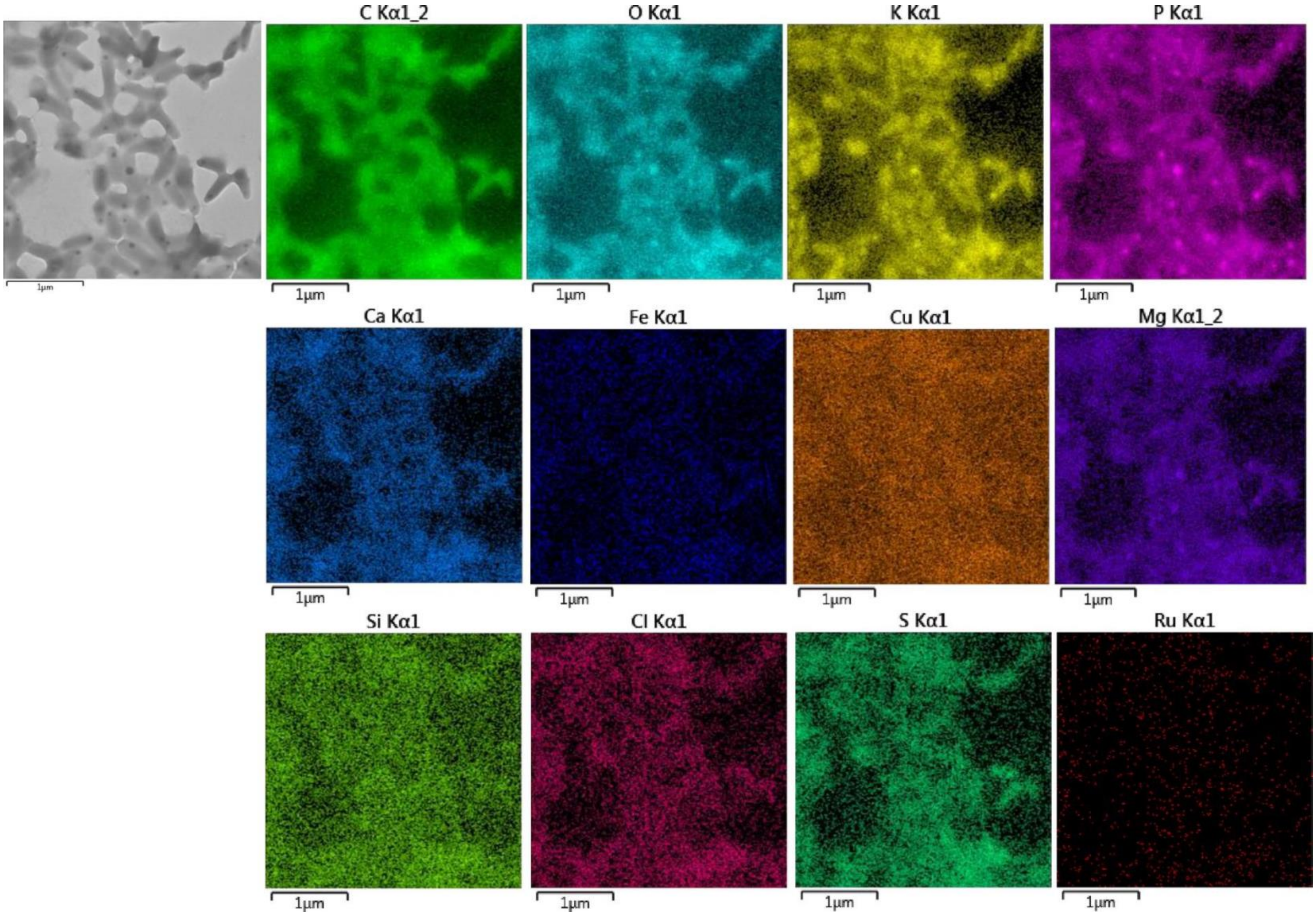

Supplementary Figure 13. TEM-EDX analysis: Images and elemental mapping of *R. cerastii* IEGM 1243 cells grown on mineral salt agar supplemented with 50 mg/L IBP for 3 days.
